# Long Peritoneal Dialysis Dwells With Icodextrin: Kinetics of Transperitoneal Fluid and Polyglucose Transport

**DOI:** 10.3389/fphys.2019.01326

**Published:** 2019-10-29

**Authors:** Anna Olszowska, Jacek Waniewski, Joanna Stachowska-Pietka, Elvia Garcia-Lopez, Bengt Lindholm, Zofia Wańkowicz

**Affiliations:** ^1^Nephrology Department, Military Institute of Medicine, Central Hospital of the Ministry of Public Defence, Warsaw, Poland; ^2^Laboratory of Mathematical Modeling of Physiological Processes, Nalecz Institute of Biocybernetics and Biomedical Engineering, Polish Academy of Sciences, Warsaw, Poland; ^3^Division of Renal Medicine and Baxter Novum, Department of Clinical Science, Intervention and Technology, Karolinska Institutet Karolinska University Hospital Huddinge, Stockholm, Sweden

**Keywords:** end-stage kidney disease, peritoneal dialysis, ultrafiltration, osmotic agent, polyglucose metabolites, amylase

## Abstract

**Background and objective:** During peritoneal dialysis (PD), the period of effective net peritoneal ultrafiltration during long dwells can be extended by using the colloidal osmotic agent icodextrin but there are few detailed studies on ultrafiltration with icodextrin solution exceeding 12 h. We analyzed kinetics of peritoneal ultrafiltration in relation to icodextrin and its metabolites for 16-h dwells with icodextrin.

**Design, setting, participants, and measurements:** In 20 clinically stable patients (mean age 54 years; 8 women; mean preceding time on PD 26 months), intraperitoneal dialysate volume (V_D_) was estimated from dilution of ^125^I-human serum albumin during 16-h dwell studies with icodextrin 7.5% solution. Sodium was measured in dialysate and plasma. In 11 patients, fractional absorption of icodextrin from dialysate, dialysate, and plasma amylase and high and low (Mw <2 kDa) Mw icodextrin fractions were analyzed.

**Results:** Average V_D_ increased linearly with no difference between transport types. At 16 h, the cumulative net ultrafiltration was 729 ± 337 ml (range −18 to 1,360 ml) and negative in only one patient. Average transcapillary ultrafiltration rate was 1.40 ± 0.36 ml/min, and peritoneal fluid absorption rate was 0.68 ± 0.38 ml/min. During 16 h, 41% of the initial mass of icodextrin was absorbed. Plasma sodium decreased from 138.7 ± 2.4 to 136.5 ± 3.0 mmol/L (*p* < 0.05). Dialysate glucose G2–G7 oligomers increased due to increase of G2–G4 metabolites while G6–G7 metabolites and higher Mw icodextrin fractions decreased. In plasma maltose and maltotriose (G2–G3 metabolites) increased while higher Mw icodextrin oligomers were almost undetectable. Dialysate amylase increased while plasma amylase decreased.

**Conclusions:** Icodextrin resulted in linear increase of V_D_ with sustained net UF lasting 16 h and with no significant difference between peritoneal transport types. In plasma, sodium and amylase declined, G2–G3 increased whereas larger icodextrin fractions were not detectable. In dialysate, icodextrin mass declined due to decrease of high Mw icodextrin fractions while low Mw metabolites, especially G2–G3, increased. The ability of icodextrin to provide sustained UF during very long dwells – which is usually not possible with glucose-based solutions – is especially important in anuric patients and in patients with fast peritoneal transport.

## Introduction

Peritoneal dialysis (PD), the leading home-based renal replacement therapy for patients with end-stage kidney disease (ESKD), associates with clinical outcomes comparable to or even better than those of in-center hemodialysis (Li et al., 2017). However, fluid overload is a common problem that is associated with poor clinical outcomes in all ESKD patients including those undergoing PD (Ng et al., 2018). Restoring water balance by adequate ultrafiltration (UF) is therefore a key target for dialysis treatment.

In PD, peritoneal transcapillary UF is typically induced using the crystalloid osmotic agent glucose and the increased dialysate tonicity results in water flow through endothelial aquaporin-1 water channels and inter-endothelial small pores into the dialysate. However, the osmotic gradient decreases due to the rapid absorption of dialysate glucose to the vascular circulation, leading to reduced and eventually negative net UF – during the overnight dwell in continuous ambulatory PD (CAPD) and the long daytime dwell in automated PD (APD) – especially in patients with fast peritoneal solute transport rate (Krediet et al., 1987; Agrawal and Nolph, 2000; Rippe and Levin, 2000; Krediet and Mujais, 2002; Qi et al., 2011; Morelle et al., 2018). Other limitations of glucose-based solutions are metabolic complications linked to glucose absorption such as hyperglycemia, hyperinsulinemia, and hyperlipidemia (Grodstein et al., 1981; Boeschoten et al., 1988) and unphysiological features (low pH, hyperosmolality, and high concentrations of lactate, glucose, and glucose degradation products) that are harmful to the peritoneal membrane (Liberek et al., 1993).

These limitations can be avoided by using the colloid osmotic agent icodextrin, which induces a more long-lasting water flow occurring predominantly through the small pores of the peritoneal capillaries (Morelle et al., 2018). Icodextrin is a mixture of starch-derived glucose polymers, linked by α1–4 (90%) and α1–6 (10%) glucosidic bonds (Alsop, 1994; Mistry, 2011), with molecular weight (MW) predominantly (>85%) ranging between 1,638 and 45,000 Daltons (Da) and only 6% having a MW less than 1,638 Da. Since the colloid osmotic pressure created by icodextrin is almost constant, UF is sustained throughout a long dwell (Mistry et al., 1987, 1994; Davies, 1994; Ho-dac-Pannekeet et al., 1996; Krediet et al., 1997; Morelle et al., 2018).

Absorption of icodextrin from the peritoneal cavity into blood is slow and occurs mainly by convective pathways *via* the lymphatics (Davies, 1994). Icodextrin is hydrolyzed in plasma by circulating intra- and extracellular α-amylase to low molecular weight (LMW) oligosaccharide metabolites, detectable in blood mainly as maltose (G2), maltotriose (G3), and maltotetraose (G4) (Mistry et al., 1994; Posthuma et al., 1997). Further metabolism of G2 is limited by the absence of maltase activity in the human circulation (Silver et al., 2014).

Compared with glucose, peritoneal UF with icodextrin is smoother with increased net UF during long dwells, particularly in fast transporters, contributing to better control of fluid balance. In addition, icodextrin may improve glycemic control, lipid profiles, phosphate removal, and cardiac function (Silver et al., 2014) and preserves residual renal function (RRF) better than glucose solutions (Chang et al., 2016).

New applications of icodextrin include initiation of PD in ESKD patients with preserved RRF using one or two icodextrin exchanges; bimodal solutions combining icodextrin with glucose in one bag for the long dwell; and using single daily long-term exchanges with icodextrin in congestive heart failure patients for treatment of overhydration and azotemia (Freida et al., 2007, 2009; Wankowicz et al., 2011; Silver et al., 2014; Kazory, 2017; Dousdampanis et al., 2018; Savenkoff et al., 2018).

Potential side-effects of icodextrin include hypotension from increased UF; loss of RRF; maltose accumulation; hypoglycemia; amylase assay interference; alkaline phosphatase increase, hyponatremia; as well as idiopathic side effects – rash, sterile peritonitis, and antibiotic compatibility (Silver et al., 2014).

The peritoneal UF can be assessed by weighing the drainage bag at the end of dialysis exchange (Silver et al., 2014) and with repeated complete drainages of the dialysate followed by reinfusions of the effluent, an intraperitoneal volume curve can be constructed (Freida et al., 2007). Another approach is to use a macromolecular volume marker to follow the kinetics of dialysate fluid volume changes intraperitoneally. This method was applied for icodextrin-based dialysis fluid using dextran 70 as a volume marker (Ho-dac-Pannekeet et al., 1996).

We have used human serum albumin labeled with iodine 125, ^125^I-HSA, a volume marker, which remains stable in both standard and alternative dialysis solutions (Baczynski et al., 2000; Marciniak et al., 2000) and applied this for detailed assessments of peritoneal transport using a thermodynamic model of solutes and water transport including comparison of glucose-based and amino acid-based solutions (Olszowska et al., 2007).

In the present study, we investigated (1) peritoneal UF during a very long (16 h) peritoneal dwell using ^125^I-HSA as intraperitoneal volume marker in clinically stable peritoneal dialysis patients and (2) kinetics of icodextrin fractions, especially LMW oligosaccharide metabolites, and the impact of these metabolites on peritoneal UF during the 16-h dwell.

## Materials and Methods

### Patients

Twenty clinically stable peritoneal dialysis patients (8 women; mean age of 54 ± 16 years) were included in the study, which was performed in years 2006–2011. Their mean body weight was 76 ± 14 kg, height 166 ± 9 cm, and residual urine volume 1,332 ± 1,111 ml/day (median 875 ml/day). The duration of preceding PD treatment was 25.7 ± 19.4 months (median 19.5 months). Seventeen patients were on CAPD and three were on APD. The causes of ESRD were chronic glomerulonephritis (11 patients), hypertensive nephropathy (2 patients), lupus nephritis (1 patient), and diabetic nephropathy (6 patients). None of the patients had peritonitis during the 3 months preceding the study. Six patients were high transporters (H), seven high-average transporters (HA), and seven low-average transporters (LA) according to peritoneal equilibration test (PET) performed 3–4 weeks before the study. In the whole group (*n* = 20), intraperitoneal dialysate volume, net ultrafiltration, and sodium concentration in dialysate and plasma were measured.

In a subgroup of 11 patients, we also analyzed fractional absorption of total icodextrin from dialysate to plasma, plasma and dialysate concentrations of amylase, plasma and dialysate LMW (Mw < 2 kDa) icodextrin metabolites comprising mainly those ranging from two glucose units (maltose, G2; Mw 360 Da) up to seven glucose units (maltoheptaose, G7; Mw 1,153 Da), and dialysate high (HMW) molecular weight icodextrin fractions. Their mean age was 50.4 ± 18.3 years (median 59 years), mean duration of PD 26.9 ± 22.4 months (median 17 months), mean body weight 73 ± 13 kg, height 164 ± 9 cm, and residual urine volume 1,113 ± 1,164 ml/day (median 700 ml/day). Three patients in this subgroup had used one exchange with icodextrin solution per day for 14.3 ± 5.1 months before the study. These patients were identified as ICO+ group and the other eight patients who did not use icodextrin before the study were identified as ICO− group (ICO-naïve patients).

The Ethics Committee of Military Institute of Medicine, Warsaw approved the study protocol. Written informed consent was obtained from each patient after an explanation of the purpose of the study.

### Study Protocol

**Figure 1:**
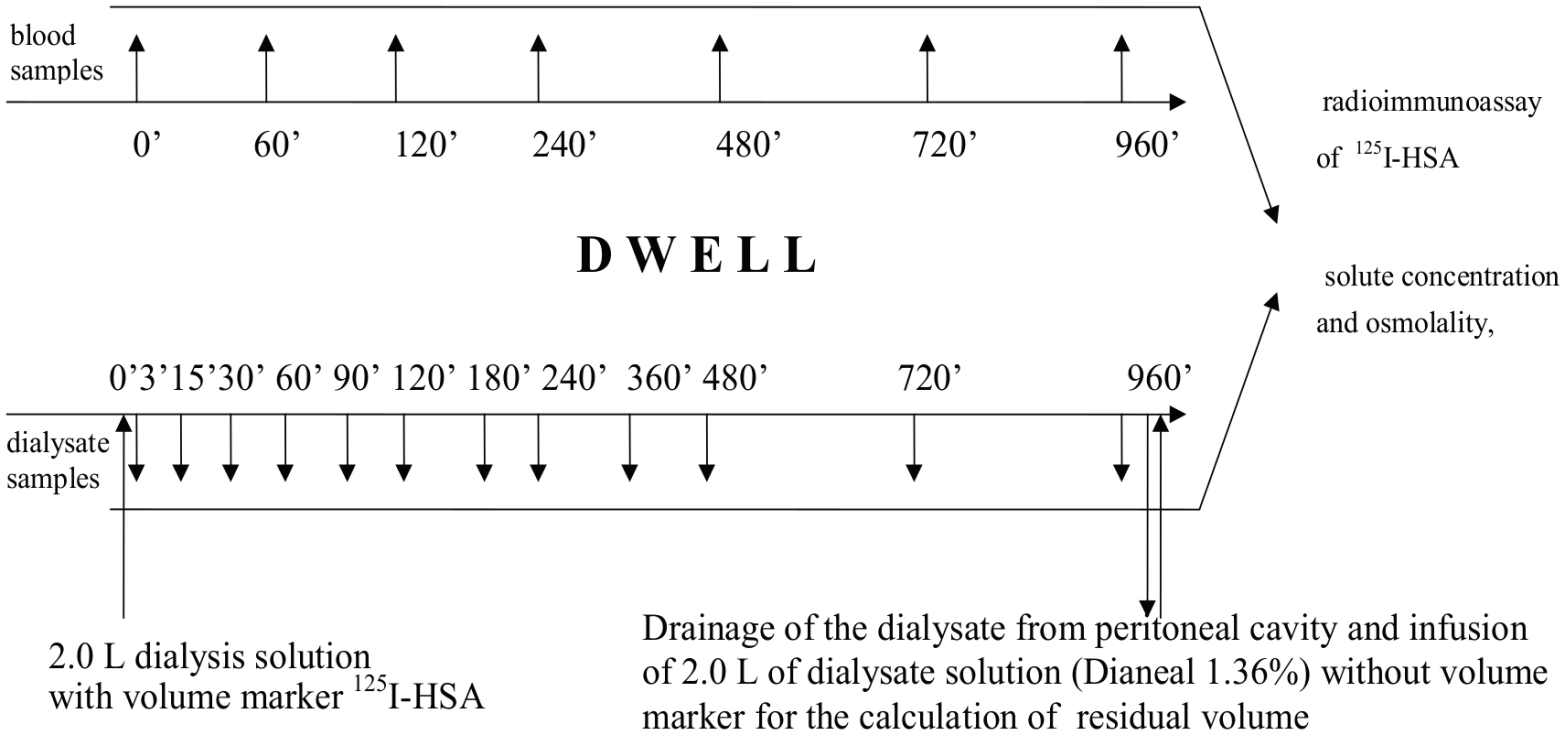
Protocol of the study.

The protocol of the study is presented in Figure 1. Immediately prior to the study, the patients received 1.0 g of vancomycin intravenously as a prophylactic against peritonitis. After the dialysate from the overnight exchange of 1.36% glucose solution had been drained from the peritoneal cavity, the patients underwent 16-h exchange with 2.0 L icodextrin dialysis fluid (Extraneal^®^, Baxter, Castlebar, Ireland). Fresh dialysis solution was pre-warmed to 37°C, and a priming dose of 0.2 g of human serum albumin (HSA) was introduced into the bag in order to minimize the adhesion of radiolabelled albumin to the surface of the plastic material. Subsequently, a “flush before fill” procedure with the new solution was performed. Radio-isotopically labeled albumin (9 mg, 7.5 μCi, ^125^I-HSA, Serlab-125; Cis Biointernational, Gif-sur-Yvette, France) was added to the dialysis solution as volume marker. After thorough mixing, the dialysis fluid was infused into the peritoneal cavity. After complete infusion, the bag was disconnected, and a three-way stopcock was placed to a transfer set. Dialysate samples (10 ml) were taken through the stopcock at 0, 3, 15, 30, 60, 90, 120, 180, 240, 480, 720, and 960 min of the exchange (Figure 1). At baseline (0 min), a sample of fresh dialysis fluid was taken from the bag when half of its content was infused. Prior to each sampling, 15 ml of the dialysate was flushed back and forth ten times through the stopcock. Immediately before collecting each sample, the patient was asked to move in order to mix the dialysate in the peritoneal cavity. Blood samples (5 ml) were collected at the 0, 15, 60, 120, 240, 480, 720, and 960 min of the exchange (Figure 1). After 960 min of peritoneal dwell, the dialysate was drained, and its volume was recorded. The peritoneal cavity was then rinsed for 5 min with 2.0 L fresh 1.36% glucose dialysis fluid without the labeled albumin to provide data for calculation of the residual peritoneal volume at the end of dwell (at 960 min). The volume of the infused fresh dialysis fluid and the volume of the drained dialysate were measured by weighing the bag and subtracting the weight of the empty bag from the full bag.

### Analytical Methods

Radioactivity of blood and dialysate samples was measured using a gamma counter (LKB Wallac1272 Clinicgamma Quatro, Turku, Finland). Sodium in plasma and dialysate was analyzed by means of a direct ion-select electrode in the hospital laboratory using Cobas Integra 760 autoanalyzer (Roche, Basel, Switzerland). Dialysate samples were analyzed for the concentration of icodextrin and its fractions as well as amylase concentration for the subgroup of 11 patients. In plasma, only LMW metabolites and amylase concentration were identified. Plasma and dialysate metabolites were measured using gel filtration high-performance liquid chromatography as described before (Garcia-Lopez et al., 2008). Plasma and dialysate α-amylase activity was determined by a fully automated routine method from Konelab 20XT routine biochemical analyzer (Thermo Clinical Labsystems Oy, Finland), using p-nitrophenol maltoheptaoside as a substrate (Garcia-Lopez et al., 2008).

### Calculations

Intraperitoneal fluid volume was estimated from the dilution of the volume marker with corrections applied for the elimination of ^125^I-HSA from the peritoneal cavity (K_E_, ml/min) and sample volumes. K_E_ was calculated as the amount of the marker absorbed from the peritoneal cavity divided by the dwell time and the average marker concentration in the dialysate during the whole dwell (Heimburger et al., 1992). Cumulative transcapillary ultrafiltration at dwell time *t* was calculated as the difference between dialysis fluid volume at time *t* and 3 min, plus cumulative fluid absorption during the same period. Net ultrafiltration at dwell time *t* was calculated as the difference between dialysis fluid volume at time *t* and 3 min.

The icodextrin concentration was considered as the sum of all high molecular weight (HMW) and low molecular (LMW) icodextrin molecules. HMW molecules of icodextrin were defined as those with molecular weight higher than that of maltoheptaose (G7, Mw 1,153 Da). Low molecular weight (LMW) metabolites of icodextrin were assessed as G2–G7 (Garcia-Lopez et al., 2008).

Peritoneal membrane transport characteristics were determined by PET as described by (Twardowski et al., 1987) and performed 3–4 weeks before the study. The data were evaluated by Student’s test. Statistical significance was accepted if *p* was less than 0.05. Results are expressed as mean ± SD.

## Results

We did not observe any clinical complications associated with the use of icodextrin solution in the 16-h dwell study and during 8 weeks of the follow-up.

### Fluid Transport

**Figure 2:**
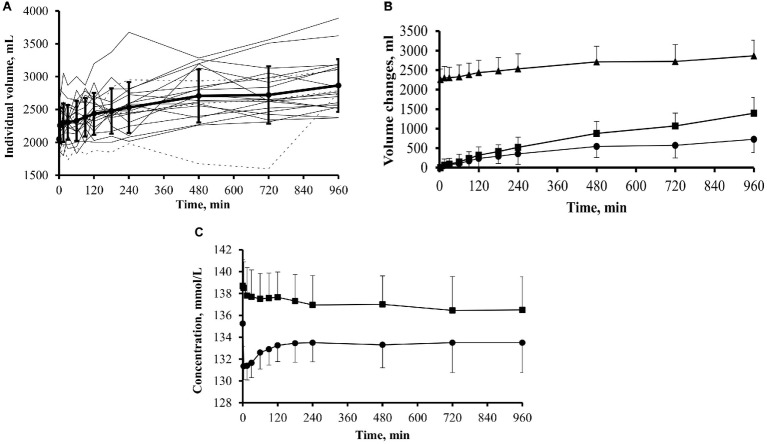
Intraperitoneal dialysate volume, net cumulative ultrafiltration, and dialysate and plasma sodium during 16-h dwells with icodextrin in 20 patients. **(A)** Individual intraperitoneal volume curves in patients previously exposed (dotted line; group ICO+; *n* = 3) and not previously exposed (solid line; group ICO−; *n* = 17) to icodextrin. **(B)** Intraperitoneal dialysate volume (▲; mean + SD), net cumulative ultrafiltration (•; mean − SD), and cumulative transcapillary ultrafiltration (■; mean + SD). **(C)** Sodium concentration in dialysate (•; mean − SD) and in plasma (■; mean + SD).

In the whole group of 20 patients, the mean intraperitoneal volume increased gradually throughout the dwell to a mean value of 2,866 ± 402 ml (range 2,372–3,621 ml) at 16 h, corresponding to a significant (*p* < 0.001) mean increase of 810 ml compared with initial values. The individual intraperitoneal volume curves (Figure 2A) showed relatively large inter-individual variations; however, in most of the patients, intraperitoneal volume increased steadily, following a linear pattern of volume increase that in 15 out of the 20 patients included the interval between 12 and 16 h. At 4, 8, 12, and 16 h of the dwell, the average net cumulative UF was 360, 546, 570, and 729 ml, respectively (Figure 2B). The mean transcapillary UF rate was 1.40 ± 0.36 ml/min. At the end of dialysis exchange, mean cumulative transcapillary UF was 1,393 ± 411 ml, and the mean cumulative net UF was 729 ± 337 ml (range from −18 to 1,360 ml). Only one patient had negative UF of −18 ml at the end of exchange. The mean peritoneal fluid absorption rate (K_E_) was 0.68 ± 0.38 ml/min. There was no difference in net ultrafiltration among the PET transport groups. For H transporters, net ultrafiltration reached 736 ± 230 ml, for HA transporters 724 ± 352, and for LA transporters 728 ± 439 ml (*p* = 1.0 for H vs. LA and HA vs. LA; *p* = 0.9 for H vs. HA). The statistical analysis of the correlation between D/P (rate of dialysate to plasma concentration) for creatinine at 4 h of the dwell with water transport (cumulative net UF as well as fluid absorption and transcapillary UF for each interval between sampling points) showed correlation only with cumulative transcapillary UF for time intervals from 15 up to 480 min and to 720 min with *r* = 0.53 (*p* = 0.016) and *r* = 0.60 (*p* = 0.006), respectively. However, the number of patients may be insufficient to draw any conclusion about the potential (and expected) impact of peritoneal solute transport rate.

The mean values of plasma concentrations of albumin and total protein (data not shown) did not correlate with the cumulative values of neither net UF, nor transcapillary UF calculated for the whole dwell period (all *p* > 0.25). Thus, the colloid osmotic pressure effect exerted by plasma albumin could not be demonstrated in the current study.

### Sodium Concentration

The concentration of sodium in dialysate decreased from 135.2 ± 2.1 mmol/L at time 0 min to 133.5 ± 2.7 mmol/L (*p* < 0.05) at the end of dwell. It declined immediately after the infusion of fresh dialysis fluid and then gradually increased up to 120 min of the dwell and thereafter remained unchanged. Plasma sodium concentration decreased from 138.7 ± 2.4 mmol/L at the start of the dwell to 136.5 ± 3.0 mmol/L at the end of the dwell (*p* < 0.05), Figure 2C.

### Kinetics of Icodextrin and Its Metabolites in ICO+ and ICO− Patients

**Figure 3:**
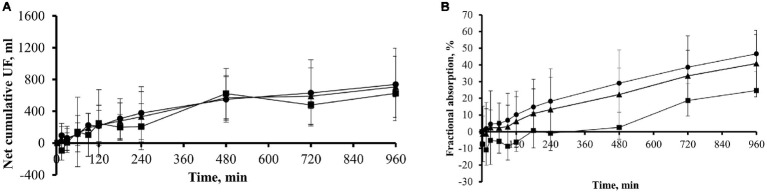
Net cumulative ultrafiltration and fractional absorbed amount of icodextrin during 16-h dwell with icodextrin in a subgroup of 11 patients. **(A)** Net cumulative ultrafiltration (mean ± SD) in subgroup of 11 patients (*n* = 11, ▲) including those previously exposed (group ICO+; *n* = 3; ■) and those not exposed (group ICO−; *n* = 8; •) to icodextrin. **(B)** Fractional (% of initial amount; mean ± SD) absorbed amount of icodextrin in subgroup of 11 patients (*n* = 11, ▲) including those previously exposed (group ICO+; *n* = 3; ■) and not previously exposed (group ICO−; *n* = 8, •) to icodextrin.

In a subgroup of 11 patients, comprising eight icodextrin naïve (ICO− group) and three icodextrin-exposed (ICO+ group) patients, the kinetics of icodextrin and its metabolites were evaluated. Net cumulative UF at the end of dwell was 706.9 ± 384.7 ml, 624.8 ± 42.3 ml in ICO+ group, and 737.7 ± 454.9 ml in ICO− group (Figure 3A).

### Dialysate Concentrations of Icodextrin Fractions and Metabolites

Icodextrin was absorbed from the dialysate to blood throughout the dwell among the eight icodextrin naïve (ICO− group) patients while – during the initial 120 min of the dwell – icodextrin was transferred in the other direction, from blood to the dialysate, in the three patients in the ICO+ group (Figure 3B). On average, about 41% of the initial intraperitoneal mass of icodextrin was absorbed during the 960 min of dwell (Table 1).

**Table 1:** Absorption of icodextrin from dialysate to plasma (mean ± SD) during 16-h dwell in subgroup of 11 patients.

Time points during the dwell (min)	Mass of absorbed icodextrin (g)	Mass of absorbed icodextrin as percentage of initial amount (%)
0	0	0
240	20.5 ± 25.8	13.3 ± 16.8
480	34.4 ± 25.8	22.3 ± 16.9
720	51.7 ± 20.7	33.4 ± 13.0
960	63.1 ± 22.9	40.8 ± 14.6

*The infused mass of icodextrin was 154.6 ± 16.6 g (mean ± SD)*.

**Figure 4:**
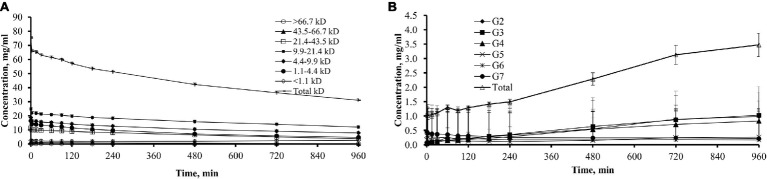
Dialysate concentrations of total icodextrin and icodextrin fractions in 11 patients. **(A)** Dialysate concentrations (mean ± SD) of total icodextrin and separate icodextrin fractions. **(B)** Dialysate concentrations (mean ± SD) of low molecular weight (LMW) icodextrin metabolites (G2–G7) and total sum of LMW icodextrin metabolites.

The pattern of changes of dialysate icodextrin concentrations differed between the different icodextrin fractions during the 16-h dwell (Figure 4). During the dwell, there was a gradual decrease of concentration of all HMW fractions in dialysate starting from 0 min of the dwell (Figure 4A). However, after a gradual decline lasting up to 240 min, the concentration of oligomers with molecular weight < 1.1 kDa increased from 480 min of the dwell and this increase continued until the end of the dwell.

The total concentration of G2–G7 oligomers in the dialysate increased significantly from 2.34 ± 1.16 mOsm/L at 0 min to 5.16 ± 2.12 mOsm/L (*p* = 0.01) at the end of the dwell (Figure 4B). From 3 min of the dwell, the concentration of G2 to G4 metabolites increased in the dialysate during the entire 16-h dwell while the concentrations of G6 and G7 metabolites decreased in dialysate throughout the whole dwell.

### Plasma Concentrations of Icodextrin Metabolites

**Figure 5:**
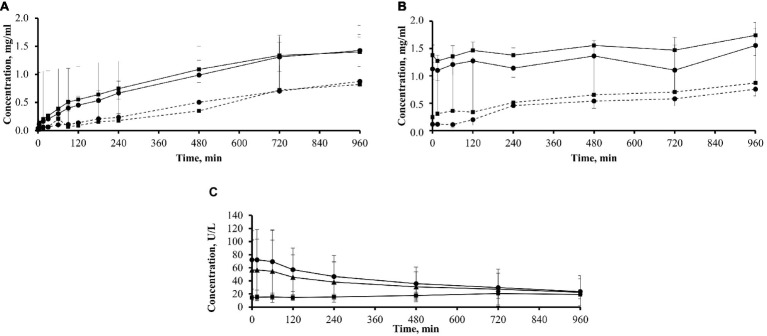
Plasma and dialysate concentrations of icodextrin metabolites G2–G3 and plasma amylase in 11 patients. **(A)** Plasma concentrations of icodextrin metabolites G2 (■; mean + SD) and G3 (•; mean − SD) in patients previously exposed (solid line; group ICO+; *n* = 3) and not previously exposed (dotted line; group ICO−; *n* = 8) to icodextrin. **(B)** Dialysate concentrations of icodextrin metabolites G2 (■; mean + SD) and G3 (•; mean − SD) in patients previously exposed (solid line; group ICO+; *n* = 3) and not previously exposed (dotted line; group ICO−; *n* = 8) to icodextrin. **(C)** Amylase concentration in plasma (mean ± SD) in a subgroup of 11 patients (*n* = 11; ▲) including those previously exposed (group ICO+; *n* = 3; ■) and not previously exposed (group ICO−; *n* = 8, •) to icodextrin during 16-h dwell with icodextrin.

The analysis of oligomer concentration in plasma was performed only for the G2–G3 metabolites since concentrations of the G4–G7 oligomers were too low to be measured in most plasma samples. In the three icodextrin-exposed patients of ICO+ group, the plasma concentrations of G2 (maltose) increased from an already high level of 1.38 ± 0.19 to 1.74 ± 0.23 mg/ml, and the concentration of G3 (maltotriose) increased from 1.12 ± 0.18 to 1.55 ± 0.19 mg/ml. In the eight icodextrin naïve patients of ICO− group, the concentration of G2 increased from 0.24 ± 0.26 to 0.87 ± 0.24 mg/ml and G3 increased from 0.11 ± 0.03 to 0.75 ± 0.12 mg/ml (Figure 5A).

### Dialysate Concentrations of Icodextrin Metabolites

In the ICO+ group (*n* = 3), the concentration of G2 increased from 0.02 ± 0.01 to 1.40 ± 0.27 mg/ml, and the concentration of G3 increased from 0.04 ± 0.02 to 1.42 ± 0.28 mg/ml (Figure 5B). In the ICO− group (*n* = 8), G2 increased from 0.01 ± 0.01 to 0.82 ± 0.55 mg/ml and G3 increased from 0.03 ± 0.02 to 0.88 ± 0.20 mg/ml.

### Amylase Concentration in Dialysate and Plasma

Amylase concentration in dialysate increased in the whole group of 11 patients from 0.61 ± 0.31 to 2.08 ± 1.40 U/L at the end of dwell (*p* < 0.01). There was a statistically significant decrease of plasma amylase concentration in the whole group during the whole dwell, from initial values of 56.43 ± 46.13 to 22.39 ± 20.57 U/L (*p* < 0.05), but the pattern of changes differed dependent on previous exposure to icodextrin (Figure 5C). In the ICO− group (*n* = 8), amylase concentration decreased from 72.14 ± 44.76 to 23.58 ± 24.20 U/L, while it increased slightly in the ICO+ group from 14.53 ± 2.72 to 19.2 ± 6.80 U/L. Plasma amylase concentration was positively correlated (at sampling points up to 480 min) with cumulative transcapillary UF calculated for initial time interval (up to 15 min) and with cumulative net UF for initial time interval (up to 15 min) and for dwell time up to 180 min: however, correlations had relatively high *p* (from *p* = 0.02 up to *p* = 0.049). The average amylase concentration in plasma correlated with cumulative net UF up to 180 min (*r* = 0.7, *p* = 0.017), and with net UF (up to 240 min, *r* = 0.63, *p* = 0.036) and cumulative transcapillary UF (up to 180 min, *r* = 0.66, *p* = 0.027). The average concentration of amylase in dialysate did not correlate with UF. The concentration of amylase in dialysate at each sampling time showed positive correlation at 15 min with cumulative UF (transcapillary UF and net UF) up to 180 and 240 min (with *r* = 0.67, *p* = 0.023 and *r* = 0.69, *p* = 0.018 for transcapillary UF and *r* = 0.73, *p* = 0.012 and *r* = 0.76, *p* = 0.006 for net UF, respectively). There was also positive correlation with *p* ≤ 0.03 of amylase level in dialysate at 60 min with net UF up to 120 min (*r* = 0.73, *p* = 0.011) and amylase level in dialysate at 90 min with net UF up to 240 min (*r* = 0.65, *p* = 0.03).

## Discussion

In our study, using a macromolecular volume marker (^125^I-HSA) to determine the intraperitoneal volume and its changes throughout the dwell, icodextrin resulted in a linear increase of net UF volume lasting up to 16 h in most of the patients. This is in contrast to the short transitory period of effective UF when glucose-based solutions are used with decline of net UF volume, starting already after 120–240 min dependent on glucose concentrations used (Heimburger et al., 1992). While the period of effective net UF is even shorter in patients with fast/high transport status when using glucose-based solutions (Heimburger et al., 1990; Wang et al., 1998b), UF patterns in the present study did not differ between patients using icodextrin solution with different peritoneal small solute transport rates, i.e., between fast/high, fast/high-average, and slow/low-average transporters.

Our findings reflect the fundamentally different molecular mechanisms and the different pathways mediating water flow between colloid osmosis induced by icodextrin versus the crystalloid aquaporin-1 dependent osmosis across the peritoneal membrane induced by glucose, as demonstrated in mice during dwells lasting 120 min (Morelle et al., 2018). Our observation that net UF with icodextrin increases linearly up to at least 16 h agrees with predictions based on computer simulations by Rippe and Levin (2000).

Although there are no previous studies that analyzed intraperitoneal dialysate volumes in patients receiving icodextrin for as long as 16 h using RISA volume marker, our results are in general agreement with some previous studies. Thus, as reported by Davies (2006), most randomized controlled clinical trials show that when using icodextrin solution, average net UF increases after 8 h of long day dwell in APD and up to 16 h of long night dwell in CAPD and this was confirmed in several recent studies (Jeloka et al., 2006; Lin et al., 2009; Takatori et al., 2011; Wang et al., 2015; Chang et al., 2016). However, results are not entirely consistent and great inter-individual variability of net UF has been reported. Jeloka et al. (2006) reported that there was no increase in net UF in patients treated by APD when their dwell time with icodextrin solution was extended from 10 h to up to 14 h; however, re-analysis of the data showed that 16 (44%) of the patients had steadily increasing UF after 10 h (Venturoli et al., 2009). Furthermore, one study did not find any statistically significant differences in UF when comparing 49 patients on ICO with 51 patients on glucose solutions for 12 months follow-up (Chang et al., 2016). Higher UF with CAPD as compared to APD has been reported in patients using icodextrin, possibly due to the increase of intraperitoneal pressure resulting from physical activity and changes in body position during the daily dwell through increase of hydrostatic capillary pressure and increase of lymphatic absorption that consequently result in lower UF (Mistry et al., 1994; Posthuma et al., 1997; Neri et al., 2000). The fact that most of the patients in our study were treated by CAPD prior to the investigation – and that most were icodextrin naïve – may have contributed to higher net UF during the dwell.

### Plasma and Dialysate Sodium in Patients Receiving Icodextrin

Icodextrin induces sustained UF even though dialysate osmolality is similar or lower than serum osmolality.

It is often assumed that the absence of free water removal when using icodextrin results in an increased convective contribution to total small solute clearance including also sodium, thereby eliminating the gap between UF and sodium removal that is seen with crystalloid osmotic agents such as glucose. We observed a statistically significant decrease in serum sodium concentration during the dialysis exchange with icodextrin; however, the concentration of sodium remained within the normal range. The decrease in serum sodium concentration is conceivably explained by dilution resulting from the increased osmotic gradient, caused by the generation of metabolites of icodextrin that induces a water flow from cells into the blood compartment. The presence of metabolites, mainly maltose (G2) and maltotriose (G3), in the vascular bed and interstitial fluid appears to have been sufficient to cause the displacement of water from cells into the extracellular space, thus resulting in hyponatremia from dilution, a phenomenon also referred to as hypertonic hyponatremia (Mistry et al., 1994; Posthuma et al., 1997; Silver et al., 2014). In patients with inadequately controlled diabetes who are treated with PD using icodextrin solution, this phenomenon could potentially add to the risk of severe symptomatic hyponatremia, resulting from inadequately controlled hyperglycemia (Silver et al., 2014).

Our results are consistent with other observations of sodium kinetics during peritoneal dwells with icodextrin based dialysis fluid. In a clinical study on 12-h peritoneal dwell with icodextrin fluid sodium concentration in serum decreased by 3 mmol/L (Moberly et al., 2002), considerably more than reported here by us. Sodium concentration in dialysate in that study increased from 131 to 134 mmol/L in 2 h and was stable throughout the following dwell time (Moberly et al., 2002), and the same pattern of increase and following stability can be found in our data although with lower amplitude of increase. After 12 h of the peritoneal dwell, the sodium concentration in dialysate was still by 5 mmol/L lower than that in plasma (Moberly et al., 2002). Experimental studies in rats demonstrated that the initial sodium concentration in dialysate (i.e., its concentration at third minute after fluid infusion) is similar for glucose and icodextrin based fluids in the control group and in the group with induced peritonitis separately (Wang et al., 2000), and the initial sodium dialysate to plasma ratio is not different if the mixture of icodextrin and glucose is applied as osmotic agent (Wang et al., 1998a). The initial sodium concentration was in the control group of animals around 131 mmol/L that was lower than the nominal concentration of sodium ions in icodextrin fluid of 133 mEq/L (Wang et al., 2000). The initial sodium concentration measured after infusion and immediate drain of icodextrin dialysis fluid in another clinical study was about 130 mmol/L (Freida et al., 2007; Galach et al., 2009). The sodium concentrations in plasma and dialysate were constant with slight tendency to decrease during 15 h peritoneal dwell (Freida et al., 2007; Galach et al., 2009). No tendency to equilibration between dialysate and plasma sodium up to 900 min of peritoneal dwell was observed (Freida et al., 2007; Galach et al., 2009). In all these studies, the sodium concentration was measured by flame photometry (Wang et al., 1998a, 2000; Freida et al., 2007; Galach et al., 2009), and in animal studies, plasma concentrations were corrected to plasma water (Waniewski et al., 1992; Wang et al., 1998a, 2000). Flame photometry measures the total sodium content in the sample and includes also the not dissociated sodium that is about 4% of total sodium, whereas our measurements performed with direct ion selective electrode report the concentration of diffusible sodium ion; the concentration of diffusible sodium ion in the study by Freida et al. might be even lower than in our study. Unfortunately, the other studies discussed here did not provide measurements in fresh dialysis fluid (Wang et al., 1998a, 2000; Freida et al., 2007; Galach et al., 2009). During dwells with glucose based solutions, sodium in dialysate is also far from equilibration with plasma sodium after 360 min in patients and, furthermore, in rats after 240 min, no sodium equilibration is observed for both glucose- and icodextrin-based fluids (Wang et al., 1997, 2000).

We have no clear explanation for the observed decrease in dialysate sodium concentration and the higher than expected initial dialysate sodium concentration. However, one possible reason for the lower than expected dialysate sodium concentration is that the measurements of sodium are somehow influenced by the presence of icodextrin, as can be observed for measurements of dialysate sodium in case of glucose-based dialysis fluid (Wang et al., 1997; La Milia et al., 2004). The observation that dialysate and plasma sodium concentrations did not fully equilibrate by 960 min is puzzling although confirmed by independent studies (Freida et al., 2007; Galach et al., 2009). One may speculate that this is due to sieving of sodium induced by LMW icodextrin fractions. Sodium concentration gradient was also maintained throughout the 6-h dwell using 3.86% glucose due to sieving of sodium in the ultrafiltrate (Wang et al., 1997).

The three pore model, which well predicted the profiles of dialysate volume change for three different fluids with glucose 3.86%, icodextrin 7.5%, and a mixture of glucose and icodextrin, was not able to correctly describe the observed lack of equilibration for sodium in plasma and dialysate for icodextrin fluid in spite of good fit to the sodium profiles in dialysate for glucose- and glucose-icodextrin-based dialysis fluids for 15-h peritoneal dwell (Galach et al., 2009). In contrast to the observed lack of equilibration in clinical data, confirmed also by our results, the modeled sodium concentration approached plasma concentration at about 4 h and remained equilibrated until 15 h (Galach et al., 2009). This failure challenges the assumptions of the three pore model as a single transport barrier between blood and peritoneal dialysate and may need a theoretical extension for polyglucose simulations as proposed for example by the spatially distributed model that involves separately two transport barriers of the capillary wall and interstitium (Stachowska-Pietka et al., 2006; Waniewski et al., 2009). One may expect higher concentration of amylase in interstitial fluid than in dialysate so the osmolality of interstitial fluid may differ from that of dialysate. According to the distributed model, the sodium dip measured for glucose-based hypertonic fluids occurs also in the interstitial fluid, not only in dialysate, and this observation suggests the importance of the processes inside the tissue in contact with peritoneal dialysate (Stachowska-Pietka et al., 2012, 2019). The conjecture about the role of interstitium in the process of osmotic fluid transport induced by polyglucose is hypothetical and will need further theoretical, experimental, and clinical studies to be confirmed.

### Kinetics of Icodextrin in Icodextrin Exposed (ICO+) and Icodextrin Naïve (ICO−) Patients

The concentration of icodextrin fractions in the fresh fluid measured in our study shows a similar pattern to that reported previously by Garcia-Lopez et al. (2005). We observed that net UF and the fractional absorption of ICO depend on the previous use of icodextrin. Among icodextrin naïve (ICO−) patients, UF was slightly higher compared to UF in icodextrin exposed (ICO+) patients (Figure 3A). This observation confirms the results of computer simulations by Rippe and Levin (2000) showing that the 7.5% icodextrin solution although slightly hypotonic to plasma, resulted in UF of 600 ml after 12-h dwell, but only in patients who were not previously exposed to icodextrin. In patients on long-term icodextrin and with steady-state plasma levels of icodextrin and its metabolites, UF after 12 h was on average 400 ml (Rippe and Levin, 2000).

The efficacy of icodextrin as osmotic agent depends on colloid osmosis, which is maintained during the long dwell due to its large size, which hinders fast absorption from the peritoneal cavity. Nevertheless, icodextrin is slowly but steadily absorbed, mainly by convective transport, from the dialysate. Because the absorption rate of icodextrin from the dialysate is almost constant during dwell, the percentage of icodextrin being absorbed is directly dependent on the duration of the exchange (Moberly et al., 2002). In our study, 22% of the initial amount of icodextrin was absorbed after 8 h and 33 and 41% after 12 and 16 h, respectively (Table 1 and Figure 3B). Other authors obtained similar results: Davies (1994) reported that the percentage of icodextrin absorbed from the dialysate was 19.6 and 33% at 8th and 16th hour, respectively. Slightly higher absorption of icodextrin was found by Moberly et al. who reported that about 40% of icodextrin was absorbed during 12-h dialysis exchanges (Moberly et al., 2002).

The evaluation of the kinetics of icodextrin during the 16-h dwell in the subgroup of 11 subjects showed a gradual decrease in the concentration of HMW fractions in the dialysate (Figure 4A). This is conceivably due to the combined effects of (1) dilution by water flow from blood, (2) intraperitoneal metabolism of the HMW fractions under the influence of α-amylase resulting in increased generation of LMW icodextrin metabolites, and (3) the absorption of these molecules from the peritoneal cavity to the peritoneal tissues, and from them through the lymphatic vessels to the blood (Moberly et al., 2002).

Intraperitoneal metabolism of icodextrin by α-amylase leads to the formation of LMW oligosaccharides, namely G2–G7 metabolites. Our study showed an increase in total LMW concentration of icodextrin metabolites in dialysate, mainly G2–G5 with a concurrent decrease in G6–G7 metabolites (Figure 4B). Moberly et al. observed a gradual decrease in the concentration of G5–G7 metabolites in the dialysate in the 12-h dwell with ICO, which they attributed to absorption of these metabolites from the peritoneal cavity into the bloodstream as well as intraperitoneal metabolism under the influence of α-amylase (Moberly et al., 2002).

The MIDAS trial, a multicenter randomized controlled study of 209 CAPD patients, compared overnight icodextrin and dextrose exchanges over a 6-month period (Mistry et al., 1994). Serum samples for estimation of icodextrin and oligosaccharides were obtained immediately prior to the study and at 1, 3, and 6 months. The mean level of icodextrin and its metabolites in plasma increased from baseline value of 0.35 g/L to a steady-state level of 4.87 g/L. The serum maltose followed an identical pattern and rose from 0.04 g/L to a steady-state level of 1.20 g/L. This increase occurred within 2 weeks of icodextrin administration, and steady-state levels were maintained throughout the 6-month study. These metabolites were not associated with any adverse clinical effects (Mistry et al., 1994). We report that the concentration of G2 and G3 metabolites in plasma and dialysate depends on the previous use of icodextrin (Figures 5A,B). The concentration of these metabolites was higher both in plasma and in dialysate in icodextrin users (average time on icodextrin solution was 14 months in the ICO+ group) compared to those not using icodextrin before the study. A similar observation was made by Davies et al. in patients receiving icodextrin for over 2 years (Silver et al., 2014); however, the levels of icodextrin and maltose fell to pretreatment values within 2 weeks after the withdrawal of icodextrin-based fluid in a small group who underwent further study. Upon recommencing icodextrin after 3 weeks period of non-use, the ICO and maltose metabolite levels rose to the initial treatment phase and reached a plateau within 2 weeks (Silver et al., 2014).

Amylase can diffuse from the blood into the dialysate, thus contributing to the metabolism of intraperitoneal icodextrin; however, intraperitoneal metabolism of icodextrin seems to be negligible when compared to the substantial transport of HMW icodextrin fractions, and LMW metabolites, from dialysate to plasma, where these fractions undergo rapid hydrolysis (Garcia-Lopez et al., 2008). In our patients, the dialysate concentration of amylase increased during the dwell but still remained low, similar to results of other studies (Garcia-Lopez et al., 2008). In contrast, the plasma concentration of amylase declined significantly in icodextrin naïve patients (ICO− group), while it remained low in the ICO+ patients conceivably related to the preceding icodextrin use (Figure 5C). A low plasma amylase concentration in patients using icodextrin may pose difficulties for the correct diagnosis of pancreatitis; however, in such patients, plasma lipase has been suggested to be of value as an alternative (Rubinstein et al., 2016). The decrease in plasma amylase activity in PD patients changing from glucose-based to icodextrin-based solutions is genuine and not only, as had been suggested by others, decreased due to interference by icodextrin in the substrates of the assay (Garcia-Lopez et al., 2008). In the current study, the baseline concentration of plasma amylase was much lower in those treated by icodextrin as compared to icodextrin naïve patients, 14.53 ± 2.72 vs. 72.14 ± 44.76 U/L.

Based on current knowledge, potential determinants of icodextrin-induced UF include: peritoneal solute transport rate, plasma colloid osmotic pressure, metabolism of icodextrin, and peritoneal clearance of macromolecules (Araujo Teixeira et al., 2002; Wiggins et al., 2005; Jeloka et al., 2006; Venturoli et al., 2009). However, some of these factors that were investigated in our study were not predictors for icodextrin-induced UF. Thus, more studies are needed to provide detailed information on the determinants for UF induced by icodextrin.

Some limitations of the study should be considered when interpreting the result. One limitation is that glucose-based solutions were not used as controls; however, the kinetics of glucose solutions have been extensively studied previously and it is unlikely that inclusion of such a control group would have changed the main conclusions. Another potential limitation is that the number of patients is relatively low; only half of the 20 patients underwent more detailed studies on the kinetics of icodextrin and its metabolites, and sodium, and only three patients had received icodextrin prior to the study. The fact that most of the patients were treated by CAPD and not by APD, and that most were not previously exposed to icodextrin should be considered when interpreting the results. Intraperitoneal pressure was not assessed in this study. Furthermore, while we estimated fluid absorption based on the elimination rate of ^125^I-HSA, our protocol did not include assessment of free water transport versus water transport through the small pores; however, it is well established that the latter is the dominating route of fluid transport when using icodextrin. Finally, the study conditions required by the complex study protocol using RISA, with need to take samples of blood and dialysate every 1 h of the dwell, deviate from the conditions of home peritoneal dialysis. On the other hand, the applied kinetic model and assessment of the transperitoneal transport of the macromolecular volume marker used in our study allowed us to determine the dynamics of UF at each time point of the 16-h dialysis exchange.

In summary, we report that the use of an icodextrin-based PD fluid resulted in an almost linear increase of intraperitoneal volume with sustained net UF lasting at least up to 16 h in most of the investigated patients and with no significant difference between peritoneal transport types. During the long dwell, plasma sodium and plasma amylase declined. The dialysate amount of icodextrin declined due to decrease of HMW icodextrin fractions while smaller LMW icodextrin metabolites, especially maltose and maltotriose, increased. Plasma maltose and maltotriose increased significantly whereas larger icodextrin fractions were not detectable in blood. The ability of icodextrin to provide sustained UF during very long dwells – which is usually not possible with glucose-based solutions – is especially important in anuric patients and in patients with fast peritoneal solute transport rates. Whether improved net UF during long-term use of icodextrin improves clinical outcomes such as technique and patient survival of PD patients depends on many other factors including residual renal function and adherence to fluid restrictions as well as other aspects of the clinical management of PD patients and will require additional studies.

## Data Availability Statement

The datasets generated for this study are available on request to the corresponding author.

## Ethics Statement

This study was carried out in accordance with the recommendations of the Ethics Committee of Military Institute of Medicine, Warsaw with written informed consent from all subjects. All subjects gave written informed consent in accordance with the Declaration of Helsinki. The protocol was approved by the Ethics Committee of Military Institute of Medicine, Warsaw.

## Author Contributions

AO, ZW, and JW designed the study in consultation with BL. AO performed and ZW supervised the clinical study. EG-L performed measurements of icodextrin metabolites. JS-P developed software for data analysis. AO and JS-P analyzed the kinetic data in consultation with JW. AO, ZW, and JW wrote the first draft. All authors revised and approved the final manuscript.

### Conflict of Interest

BL is employed by Baxter Healthcare at Baxter Novum, Karolinska Institutet.

The remaining authors declare that the research was conducted in the absence of any commercial or financial relationships that could be construed as a potential conflict of interest.
